# Research on product detection and recognition methods for intelligent vending machines

**DOI:** 10.3389/fnins.2023.1288908

**Published:** 2023-11-13

**Authors:** Jianqiao Xu, Zhifeng Chen, Wei Fu

**Affiliations:** ^1^Department of Information Security, Naval University of Engineering, Wuhan, China; ^2^School of Computer Science and Artificial Intelligence, Wuhan University of Technology, Wuhan, China

**Keywords:** deep learning, computer vision, object detection, ResNet, intelligent vending machines

## Abstract

With the continuous development of China's economy and the improvement of residents' living standards, it also brings increasing costs of labor and rent. In addition, the impact of the pandemic on the entity industry has brought opportunities for the development of new retail models. Based on the booming development of artificial intelligence, big data, and mobile payment in the new era, the new retail industry using artificial intelligence technology has shown outstanding performance in the market. Among them, intelligent vending machines have emerged in the new retail model. In order to provide users with a good shopping experience, the product detection speed and accuracy of intelligent vending machines must be high enough. We adopt Faster R-CNN, a mature object detection algorithm in deep learning, to solve the commodity settlement scenario of intelligent vending machines.

## 1. Introduction

In recent years, deep learning-based computer vision methods have received extensive research attention, especially the ResNet proposed by He et al. (2016), which addressed the degradation problem caused by increasing the number of layers in neural networks, and the Faster R-CNN proposed by Ren et al. (2017), which has made significant progress in object detection. These mature and efficient artificial intelligence algorithms have been widely used in the new retail industry, such as intelligent vending machines that use computer vision algorithms discussed in this article. Compared with traditional vending machines or physical retail stores, intelligent vending machines have lower costs, more flexible types of goods sold, and higher profits to the retail industry, thus standing out in the new retail market.

The object detection of retail product checkout in intelligent vending machines faces several challenges. One challenge is that it is difficult to predict user behavior, and the products in checkout images may be stacked, placed in abnormal ways, or obscured by obstacles (such as hands). The challenges mentioned above may result in the algorithm receiving insufficient information. Therefore, it is essential to ensure that the accuracy of product detection meets the requirements in such cases. Another challenge is the detection speed of the algorithm, which is crucial for improving the user experience. This article addresses these two issues by selecting a unique dataset for training on single-target commodities from multiple angles and perspectives and verifying it on multi-target items. Meanwhile, ResNet50 is chosen as the backbone neural network of Faster R-CNN to improve feature extraction for each product's angle and enhance the overall performance and prediction speed of the model. The Faster R-CNN based on ResNet50 used in this article achieves good accuracy and acceptable response speed in the intelligent vending machine product checkout scene.

## 2. Related work

Intelligent vending machines have advantages such as flexible stocking and low costs compared to traditional vending machines. Classic vending machines have complex manufacturing processes and high prices, are limited by the structure of the vending channel, and have a specific failure rate. At present, there are three different technical solutions for intelligent vending machines, namely gravity induction (Brolin et al., 2018), radio frequency identification (RFID) (Ramzan et al., 2017), and computer vision algorithms based on deep learning. The gravity solution is just an improvement method for traditional vending machines, and it does not entirely overcome the shortcomings of conventional vending machines. Due to technical limitations, RFID cannot perform well on metal goods, meaning that canned beverages are unsuitable for RFID vending machines. Moreover, because of the technical characteristics of RFID, each item must be manually labeled with an RFID tag before being placed in the intelligent vending machine for sale; this is an additional cost that cannot be ignored for the RFID technical solution.

The fundamental technology of intelligent vending machines based on computer vision is to identify products through images captured by the camera. Many works have achieved significant success in object detection (Li et al., 2016; Nian et al., 2016; Zhang et al., 2019; Ren et al., 2020, 2022), which can be applied to product recognition. Currently, deep learning-based object detection algorithms have become mainstream. These algorithms can be divided into two main types: region proposal-based methods and single-stage methods. Region proposal-based methods generate candidate regions and then classify and regress these regions to obtain the final detection results. These methods include RCNN (Girshick et al., 2014), Fast RCNN (Girshick, 2015), Faster RCNN (Ren et al., 2017), etc. Single-stage methods directly classify and regress the image without generating candidate regions. These methods include YOLO (Redmon et al., 2016; Redmon and Farhadi, 2017, 2018; Bochkovskiy et al., 2020), SSD (Liu et al., 2016), RetinaNet (Lin et al., 2017), etc. In addition, object detection faces many challenges, such as occlusion, scale variation, and illumination variation. To overcome these challenges, researchers have proposed many improved algorithms. For example, Mask RCNN (He et al., 2017) adds instance segmentation functionality to Faster RCNN, allowing the model to detect and segment objects simultaneously. CenterNet (Zhou et al., 2019) is a center point-based detection algorithm that can maintain high accuracy while improving detection speed.

For the datasets of retail product checkout, Goldman (Goldman et al., 2019) assembled a dataset consisting of images of supermarket shelves. It contains 110,712 product categories, averaging 147.2 instances per image. The dataset we used, Retail Product Checkout (RPC) proposed by Wei et al. (2019), is a large-scale retail dataset that includes 83,739 images with bounding box annotations for 200 categories of products. In the PRC dataset, training images only contain a single object. In contrast, testing images may contain multiple objects and are divided into three groups: easy, medium, and hard, making it an ideal dataset for our purposes.

## 3. Product detection and recognition methods for intelligent vending machines

**Figure 1:**
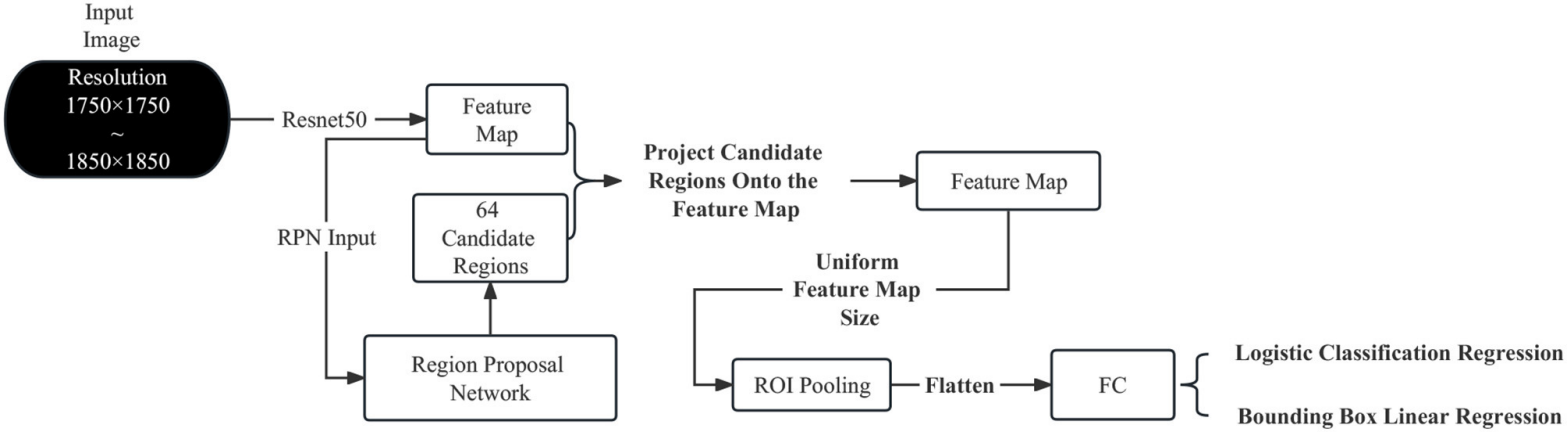
Network architecture of the faster R-CNN based on ResNet50.

This section applies the Faster R-CNN to the product settlement scenario of intelligent vending machines. Figure 1 illustrates the network architecture of the Faster R-CNN based on ResNet50, which can be summarized as the RPN network + Fast R-CNN. In this network, the candidate regions for Fast R-CNN are not selected by the Selective Search algorithm (Uijlings et al., 2013) but are provided by the RPN. Additionally, the Faster R-CNN used in this paper extracts features from the input image using ResNet50 rather than VGG16.

### 3.1. Input image preprocessing

The input image resolution of the dataset used in this paper ranges from 1,750 × 1,750 to 1,850 × 1,850. High-resolution images provide more detailed information but pose challenges for training due to the large number of parameters and calculations required by the deep neural network ResNet50 used in this paper. Modern deep-learning methods commonly use GPU acceleration for training. Still, training on personal computers with limited GPU and memory resources can easily lead to memory overflow and out-of-memory errors. For example, on my personal computer with 16GB RAM and 8GB GPU memory, when the batch size is set to 3, the memory usage is up to 95% when using the Dataloader to read data, and the GPU memory overflow occurs when preparing to start training after reading the data. When the batch size is set to 2, the training time for one epoch is as long as eight hours. Therefore, we attempted to reduce the resolution of all input images from 3 × 438 × 438 to 3 × 463 × 463 before training. And when calculating the bounding box loss, the predicted coordinates of the model's bounding boxes are multiplied by four before being compared to the coordinates in the labels. This can be done because there are generally no tiny targets in the checkout scenario, so the negative impact on the model is relatively small. Through experiments, this has been shown to improve the training speed.

### 3.2. ResNet50

**Figure 2:**
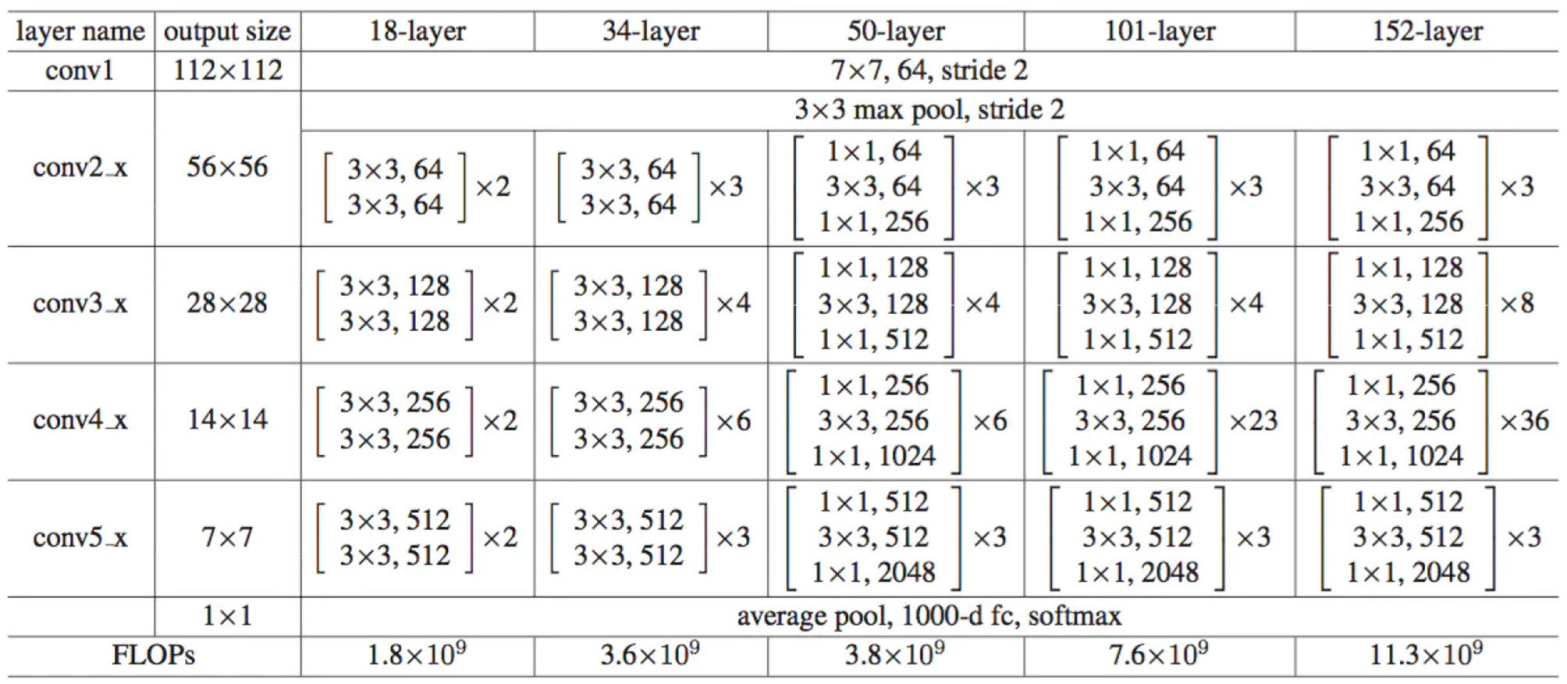
Network architecture of ResNet.

As shown in Figure 2, the first layer of all ResNet consists of a 7 × 7 convolutional layer with a stride of 2, followed by a 3 × 3 max pooling layer with a stride of 2. After the convolutional layer, there is a 3 × 3 max pooling layer with a stride of 2. The max pooling layer downsamples the feature maps output from the convolutional layer, reducing the size of the feature maps while retaining the most salient features. After passing through the common convolutional and pooling layers, all ResNet structures are followed by four residual block layers. Specifically, implementing the residual block in ResNet involves adding a shortcut connection between two convolutional layers and adding the input directly to the output of the convolutional layers.

When VGG16 was used as the backbone neural network in the original Faster R-CNN paper, the number of parameters used for feature extraction was ~ 138 M, with a floating-point calculation of 30.8 G FlOPS. In contrast, ResNet50 only had about 23 M parameters and 8.2 G FlOPS floating point calculations. During training, ResNet50 had a much faster convergence rate than VGG16, making it both quick and efficient, significantly reducing training time. Additionally, ResNet50 has a larger receptive field in its feature map than VGG16 due to the multiple convolutional layers, which allows it to capture larger image contexts. A larger receptivefield is generally better in object detection tasks, as it can capture more overall features. When the receptive field is not large enough, it can cause the model to have bias errors, seriously affecting its performance. ResNet50 has a receptive lot of approximately 483, while VGG16's receptive field is only 212. Since the target pixels in the images used in this paper are mostly equal to or larger than 300 × 300, ResNet50 is better suited to this task than VGG16. The formula for calculating the receptive field is as follows:


(1) $$\begin{array}{l} RF_{i}=\left(RF_{i-1}-1\right)*Stride_{i}+K_{sizei} \end{array}$$

*RF*_*i*_ refers to the receptive field of the i-th layer; *Stride*_*i*_ is the stride of the i-th layer; *K*_*sizei*_ is the size of the convolutional kernel used in the i-th layer.

### 3.3. Faster R-CNN

#### 3.3.1. Region proposal network

In Faster R-CNN, the role of the region proposal network(RPN) is to generate region proposals, which are candidate regions that may contain objects. These region proposals are then fed into a subsequent classification network for object detection.

**Figure 3:**
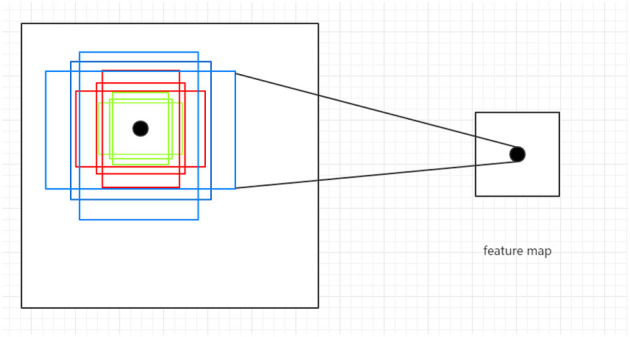
Illustration of anchor boxes.

The RPN operates on a feature map and uses a convolutional neural network over the feature map, generating multiple anchor boxes of different sizes and aspect ratios, as shown in Figure 3. There are three sizes of anchor boxes, which are 128, 256, and 512, and three aspect ratios, which are 1:2, 2:1, and 1:1. Based on the combinations of sizes and aspect ratios, nine different anchor boxes are generated at each point in the feature map, with their coordinates projected onto the original image as the center. For each anchor box, the RPN predicts whether it contains an object and the rough location of the object, thus generating region proposals. These region proposals can then be fed into a subsequent classification network for object detection, resulting in the final detection results. In the end, we divided the image into 9 × 14 × 14 anchor boxes (approximately 1.7k). Some of the anchor boxes we split may span across boundaries, but we ignore those that do. After removing the anchor boxes that span across boundaries, we sample 64 anchor boxes from the remaining ones, with an equal distribution of positive and negative samples, each accounting for 50%. If there are not enough positive samples to fill half of the selected samples, we can use negative samples to fill the remaining slots. Whether the IoU (Intersection over Union) value between each candidate box^1^ and the ground-truth box exceeds a preset threshold is the criterion for determining positive and negative samples.

The loss function of RPN consists of two parts: classification loss and bounding box regression loss. In the classification loss function, we calculate a binary classification loss for each anchor box, representing the error of classifying it as foreground (containing an object) or background (not including an object). For each anchor box, the corresponding binary classification loss is:


(2) $$L_{cls}=\left\{ \begin{array}{l} -log(p)\;if(y==1)\\ -log(1-p)\;else \end{array}\right.$$

Where *p* represents the predicted probability of the anchor box being classified as foreground, *y* represents the ture label.When *y* = =1, it represents true lable of the anchor box is foreground, and when *y* = =0, the true label is the background. In the bounding box regression loss function, we calculate a smooth L1 loss for each anchor box that is classified as foreground, which represents the difference between the predicted bounding box coordinates and the true bounding box coordinates. For each foreground anchor box, its corresponding L1 loss is:


(3) $$smooth_{L1}(x)=\left\{ \begin{array}{l} 0.5*x^{2}\;if(x<1)\\ |x|-0.5\;else \end{array}\right.$$

(4) $$\begin{array}{l} L_{reg}\left(t^{*},t\right)=smooth_{L1}\left(t_{i}^{*}-t_{i}\right) \end{array}$$

Here, t^*^ represents the true bounding box coordinate offset, *t* represents the predicted bounding box coordinate offset, and *i* represents the dimension of the coordinate axis. The *N* represents the number of anchor boxes classified as foreground.After computing the loss functions for both components, we add them together to obtain the final RPN network loss function:


(5) $$\begin{array}{l} L_{RPN}=\frac{1}{N_{cls}}\overset{N_{cls}}{\underset{i}{\sum }}L_{cls}+\lambda \frac{1}{N_{reg}}\overset{N_{reg}}{\underset{i}{\sum }}p_{i}L_{reg}\left(t_{i}^{*},t_{i}\right) \end{array}$$

*p* and *t* denote the classification prediction and bounding box regression prediction of the RPN network, while and t^*^ represent the true bounding box coordinate offsets. *N*_*cls*_ and *N*_*reg*_ correspond to the numbers of all and foreground anchor boxes, respectively. λ is a hyperparameter that balances the classification loss and bounding box regression loss.

#### 3.3.2. ROI pooling

Since the dimensions of the images are not the same, it means that the corresponding feature map sizes are also different. The purpose of ROI pooling is to unify the feature map sizes, making it easier for subsequent neural network processing. The implementation of ROI pooling involves dividing the feature map into 7 × 7 regions and performing max pooling within each region.The feature map image outputted by the ROI Pooling layer is a three-dimensional tensor of size 7 × 7 × 2048. We flatten it into a one-dimensional vector of size 1 × 100,352. Then, we concatenate these vectors in the order of their corresponding ROIs in the input image, forming a two-dimensional tensor of size 64 × 100,352 This two-dimensional tensor serves as the input to the fully connected layer for classification and regression tasks.

## 4. Experimental analysis

### 4.1. Retail product checkout dataset introduction

The dataset used in this article is a large-scale retail product checkout dataset publicly available on Kaggle (link: https://www.kaggle.com/datasets/diyer22/retail-product-checkout-dataset). This dataset provides rich image data of products during the checkout process and is currently the largest dataset regarding the number of images and product categories. It includes 200 common product categories in daily life, with a training set of 48,000 single-product images, a test set of 24,000 multi-target product images, and a validation set of 6,000 multi-target product images.

**Figure 4:**
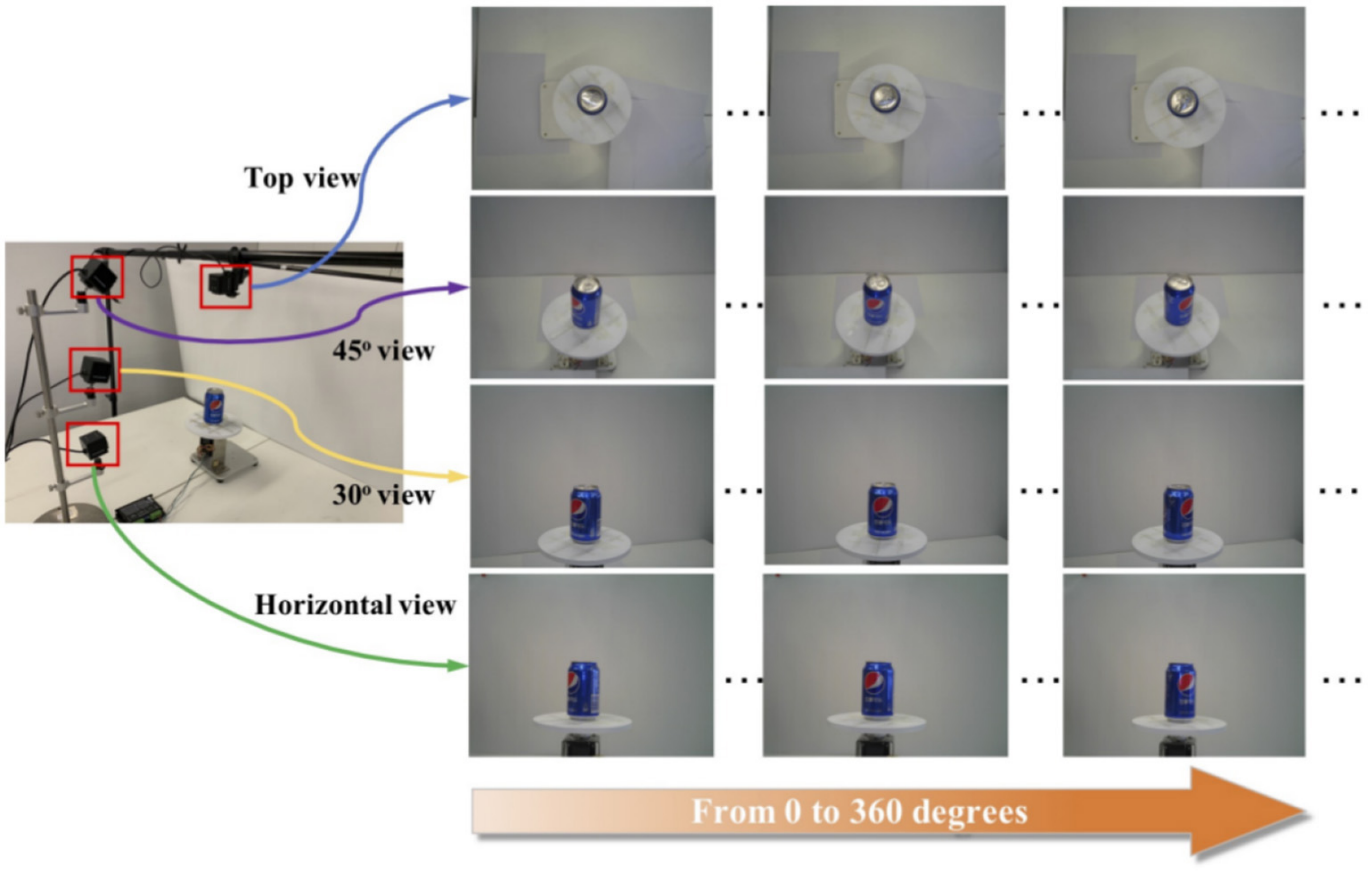
The collection form of the training set.

The training set consists of single-object images captured by four cameras placed at the top, 45 degrees upward, 30 degrees upward, and horizontally in a specified environment, covering 0–360 degrees, as shown in Figure 4. The validation and test sets are multi-object images. They are categorized into easy mode, medium mode, and hard mode based on the clutter level of the products in the images. The training set consists of single-object images captured by four cameras placed at the top, 45 degrees upward, 30 degrees upward, and horizontally, respectively, in a specified environment, covering 0–360 degrees. The validation and test sets are multi-object images and are categorized into easy mode, medium mode, and hard mode based on the clutter level of the products in the images.

**Figure 5:**
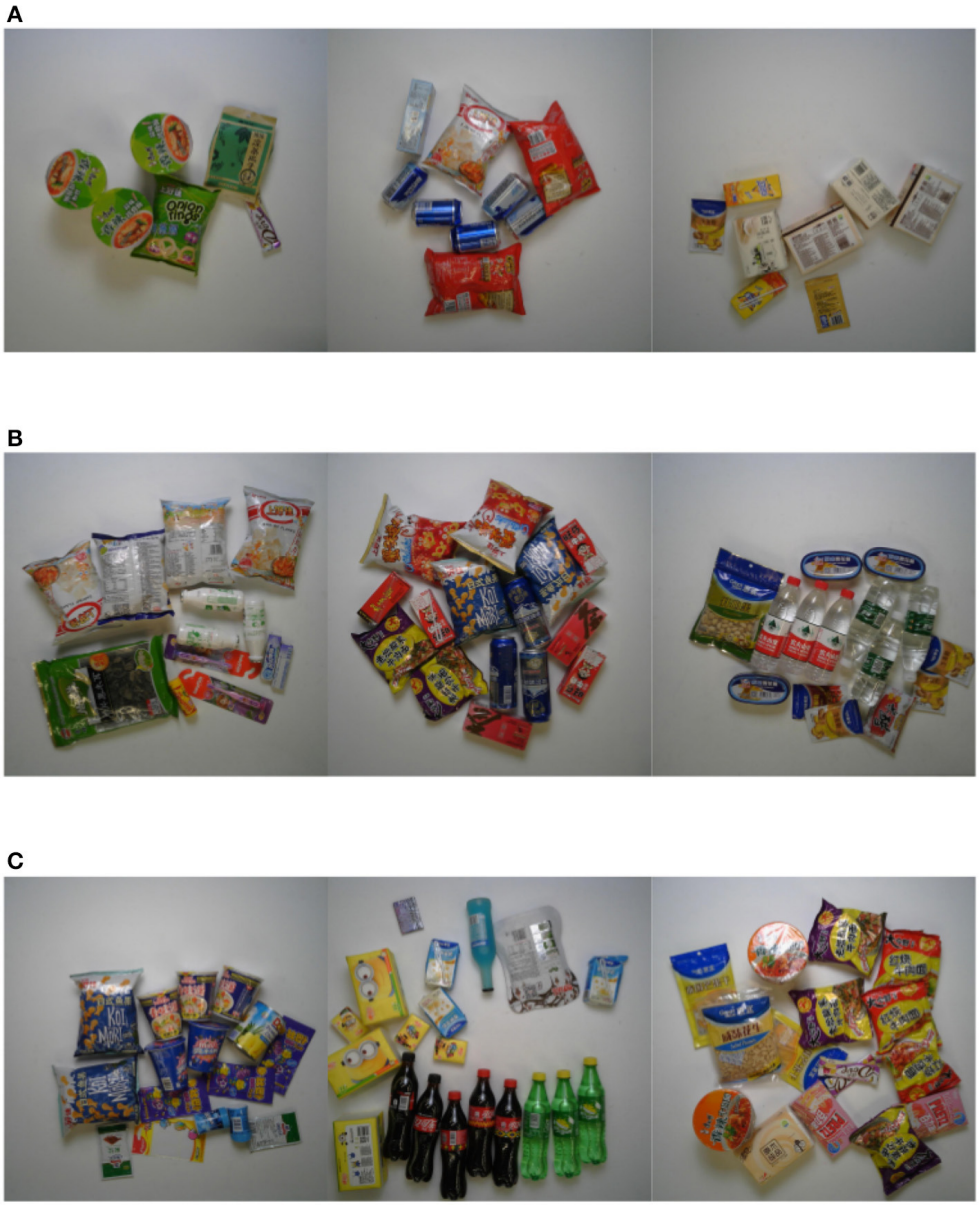
Three levels of validation difficulty. **(A)** Easy mode. **(B)** Medium mode. **(C)** Hard mode.

The dataset validation is divided into three levels of difficulty based on the complexity of product arrangement, as shown in Figure 5.

### 4.2. Experimental parameter settings and experimental environment

The experimental environment is a personal computer with the following specifications: Processor: AMD R7-5800H; GPU: NVIDIA RTX 3070 8G; Memory: 16G. The editor used is Pycharm 2022.1; operating system: WIN11; CUDA version: 11.02; Pytorch version: 1.11.0. We used the Pytorch framework to construct our model. Before starting the training, we loaded the pre-trained parameters of ResNet50 into the model to speed up the training process. The optimizer we used is the stochastic gradient descent algorithm with a momentum value of 0.9 and set weight decay to prevent overfitting. Finally, we set a learning rate with dynamic decay. Since we trained on a personal computer with limited GPU memory, we set the batch size to 4.

### 4.3. Analysis of experimental results

In order to evaluate the model we trained, we used Pycocotools provided by the COCO official for evaluation. It provides 10 evaluation metrics including AP (Average Precision), AP (IOU = 0.5), AP (IOU = 0.75), AP (Small Area), AP (Medium Area), and AP (Large Area), AR (Average Recall), AR (Max = 1), AR (Max = 10), and AR (Max = 100). Among them, AP(IOU = 0.5) is the most commonly used metric. The experimental results are shown in Table 1. The above results indicate that using the Faster R-CNN algorithm for object detection on the Retail Product Checkout dataset can achieve good performance. The performance of the model varies under different AP metrics, with AP (IOU = 0.5) and AP (Large Area) performing well and AP (Small Area) and AP (Medium Area) performing poorly. This is because the environment of the intelligent vending machine is relatively fixed, and there are no small or medium-sized objects in the dataset, so APs and APm are close to 0. This can also be inferred from the fact that APl and AP values are always close.

**Table 1:** Evaluation results.

**Epoch**	**Average precision**					
	**AP**	**AP (IOU = 0.5)**	**AP (IOU = 0.75)**	**AP (Small area)**	**AP (Medium area)**	**AP (Large area)**
20	0.539	0.6379	0.5596	0	0	0.5391
24	0.5794	0.6412	0.5784	0	0	0.5795
28	0.5818	0.6415	0.5807	0	0	0.5819
32	0.5888	0.6435	0.5825	0	0	0.5986
36	0.5962	0.6463	0.5875	0	0	0.5972
40	0.5921	0.6484	0.5866	0	0	0.5921

### 4.4. Detection performance of the model under different difficulty levels

**Figure 6:**
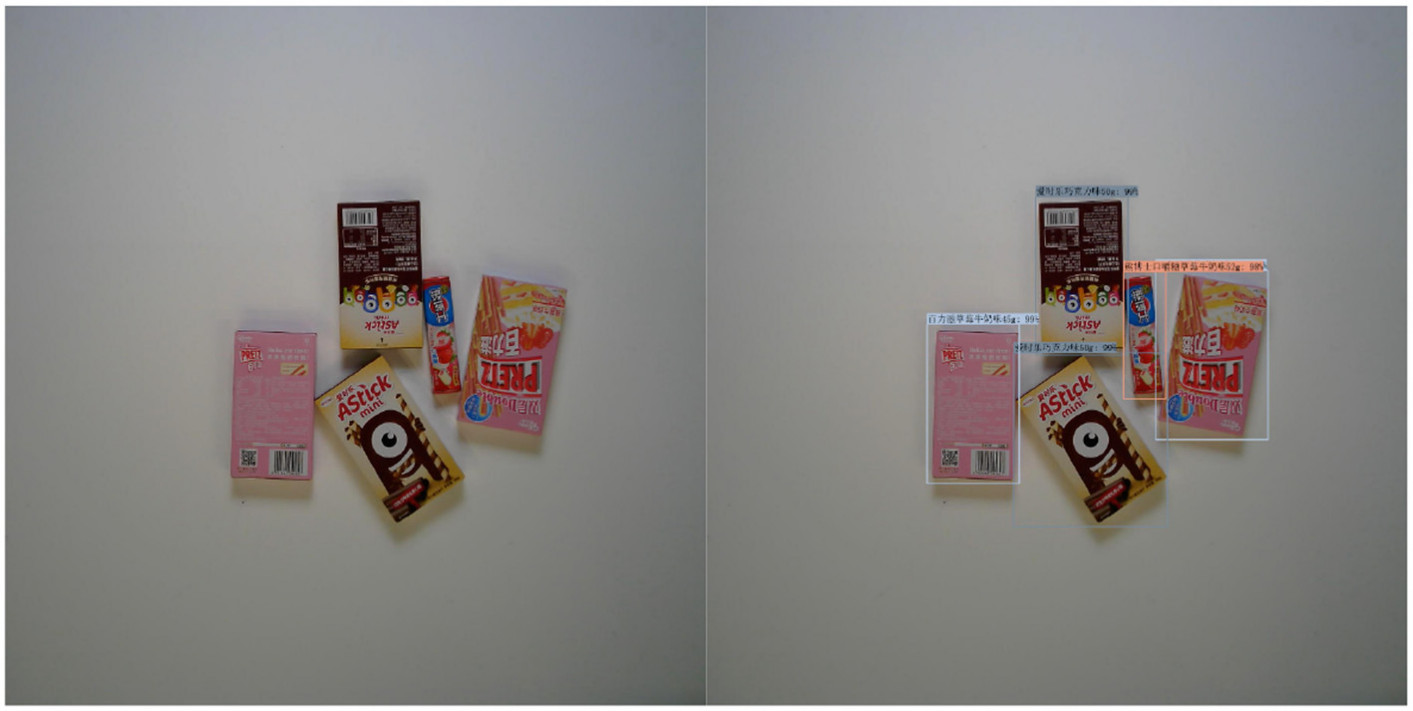
Easy mode.

**Figure 7:**
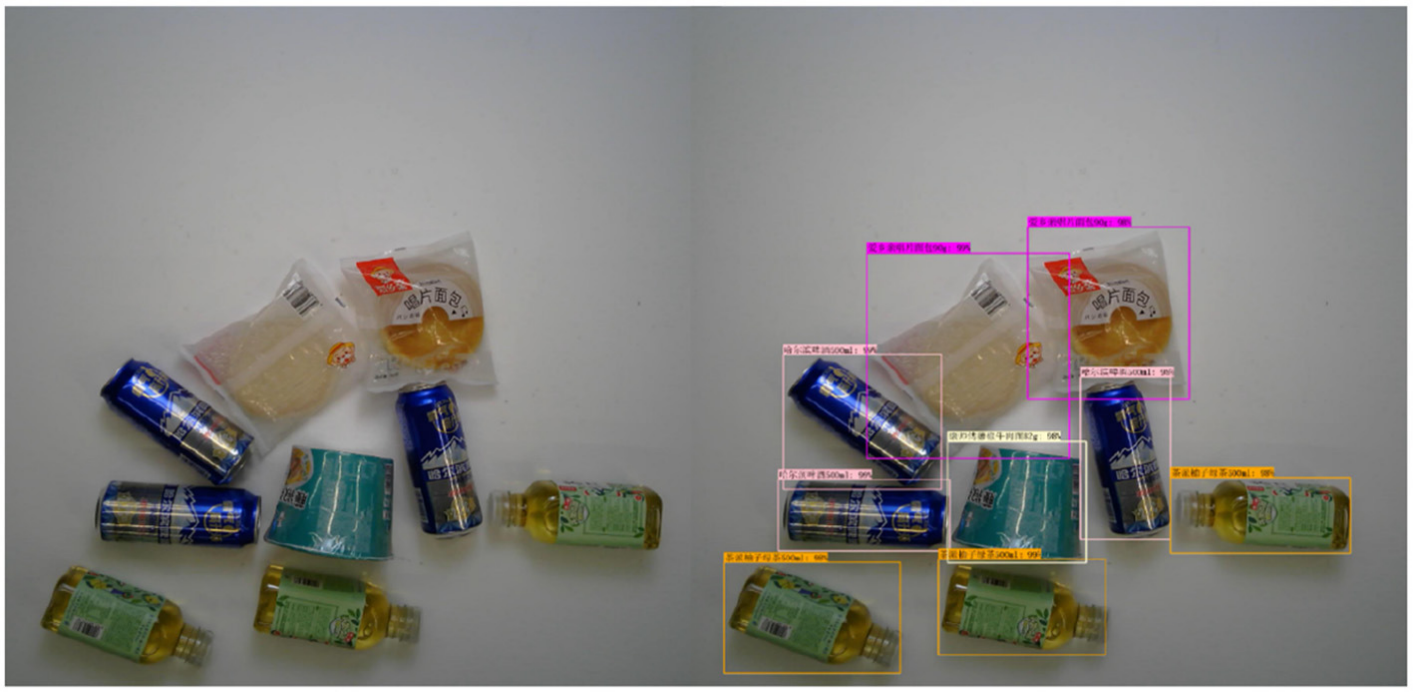
Medium mode.

**Figure 8:**
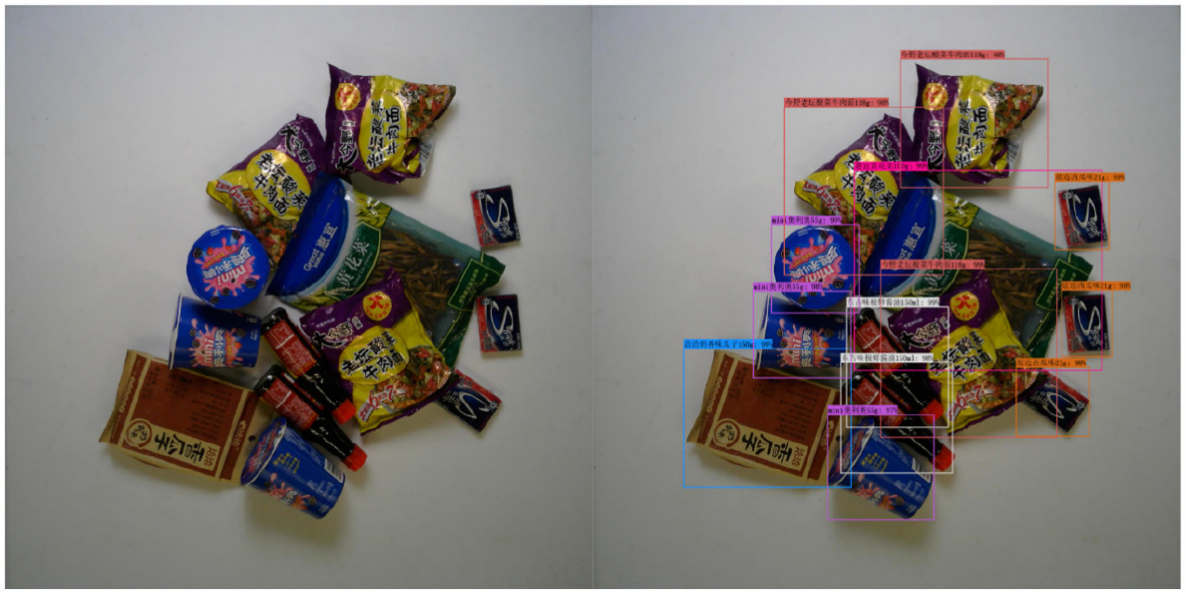
Hard mode.

This section presents the model's prediction performance under different difficulty levels, and the detection of the goods is good. See Figures 6–8.

## 5. Conclusion

After years of development, object detection technology has made rapid progress, and there are now many mature and efficient object detection algorithms such as Faster R-CNN, YOLO, SSD, and others. In this paper, we successfully applied Faster R-CNN for object detection in the context of commodity settlement and achieved good results. Using object detection in computer vision as a commodity settlement recognition task for intelligent vending machines is reliable, low-cost, and efficient.

The Faster R-CNN object detection model based on ResNet50 constructed in this paper achieved good results on a large commodity dataset, with precision meeting the requirements on recognized targets and a very low probability of misclassification. However, there are still cases of missed detections in multi-object scenarios, which I believe can be improved through further training. At the same time, the model constructed in this paper has already met the recognition speed requirements for intelligent vending machines, but there is still room for improvement.

## Data availability statement

Publicly available datasets were analyzed in this study. This data can be found here: https://www.kaggle.com/datasets/diyer22/retail-product-checkout-dataset.

## Author contributions

JX: Conceptualization, Investigation, Software, Writing—original draft, Writing—review & editing. ZC: Writing—original draft, Writing—review & editing. WF: Writing—review & editing, Funding acquisition.

## Footnotes

^1^The sampled 64 anchor boxes are referred to as candidate boxes.
